# Diagnostic Performance of Fas Ligand mRNA Expression for Acute Rejection after Kidney Transplantation: A Systematic Review and Meta-Analysis

**DOI:** 10.1371/journal.pone.0165628

**Published:** 2016-11-03

**Authors:** Baoli Heng, Hongwen Ding, Haolin Ren, Liping Shi, Jie Chen, Xun Wu, Caiyong Lai, Ganshen Yu, Yin Xu, Zexuan Su

**Affiliations:** Department of Urology, the First Affiliated Hospital of Jinan University, Guangzhou, China; Icahn School of Medicine at Mount Sinai, UNITED STATES

## Abstract

**Background:**

The value of Fas ligand (FASL) as a diagnostic immune marker for acute renal rejection is controversial; this meta-analysis aimed to clarify the role of FASL in acute renal rejection.

**Methods:**

The relevant literature was included by systematic searching the MEDLINE, EMBASE, and Cochrane Library databases. Accuracy data for acute rejection (AR) and potential confounding variables (the year of publication, area, sample source, quantitative techniques, housekeeping genes, fluorescence staining, sample collection time post-renal transplantation, and clinical classification of AR) were extracted after carefully reviewing the studies. Data were analyzed by Meta-DiSc 1.4, RevMan 5.0, and the Midas module in Stata 11.0 software.

**Results:**

Twelve relevant studies involving 496 subjects were included. The overall pooled sensitivity, specificity, positive likelihood ratio (LR), negative LR, and diagnostic odds ratio, together with the 95% CI were 0.64 (0.57–0.70), 0.90 (0.85–0.93), 5.66 (3.51–9.11), 0.30 (0.16–0.54), and 30.63 (14.67–63.92), respectively. The area under the summary receiver operating characteristic curve (AUC) was 0.9389. Fagan’s nomogram showed that the probability of AR episodes in the kidney transplant recipient increased from 15% to 69% when FASL was positive, and was reduced to 4% when FASL was negative. No threshold effect, sensitivity analyses, meta-regression, and subgroup analyses based on the potential variables had a significant statistical change for heterogeneity.

**Conclusions:**

Current evidence suggests the diagnostic potential for FASL mRNA detection as a reliable immune marker for AR in renal allograft recipients. Further large, multicenter, prospective studies are needed to validate the power of this test marker in the non-invasive diagnosis of AR after renal transplantation.

## Introduction

Paralleling the leveling off of the incidence of end-stage renal disease, the number of kidney transplants has remained stable since 2005. More and more patients live with a functioning kidney transplant [1]. Attention continues to be focused on reducing acute rejection (AR) and other post-transplant complications, and on improving long-term outcomes. Recent reports have shown that the incidence of AR affects 10%–15% of patients in the first year post-transplant, depending on the immunosuppressive strategy[2, 3], and has declined > 50% since 2000, but has remained stable in the past 5 years [1]. Indeed, AR episodes remain a strong predictor of graft failure. Recent reports have shown that all types of acute cellular rejection (ACR) according to the Banff classification can influence the long-term survival of allografts, and vascular or late ACR can predict poorer graft survival[4]. Furthermore, for low-risk recipients, excessive immunosuppression should be avoided in clinical practice because of the major adverse effects[5].

Currently, monitoring strategies for post-renal transplantation include serial serum creatinine detection, clinical follow-up, and in some programs, protocol biopsies, none of which predict AR [6]. Moreover, as the gold standard for diagnosing AR, kidney allograft biopsy still has limitations, such as the risk of bleeding, adjacent organ injury, and misdiagnosis caused by possible sampling error[7–9]. In the past three decades, clinicians have sought non-invasive tools by which to identify AR early and replace the need for kidney biopsy [10].

Immune monitoring has been suggested as a promising strategy that may detect initial inflammatory changes in the allograft and lead to early therapeutic intervention [6]. Because the distinctive feature of allograft rejection is infiltration by T lymphocytes, markers related to cytotoxic T lymphocytes (CTLs) may be useful in monitoring AR episodes. Fas ligand (FASL, FASLG, APTL, CD178, CD95L, ALPS1B, CD95-L, APT1LG1, TNFSF6, and TNLG1A) is the key death factor of receptor-triggered programmed cell death in immune cells, and also one of the major effector molecules of CTLs during the AR response. The Fas-FASL interaction and apoptosis are important in the mechanism underlying allograft rejection [11]. Sharma et al. [12]first identified intrarenal expression of FASL messenger RNA (mRNA) using a real-time polymerase chain reaction (quantitative [q]PCR) technique, and suggested that the intrarenal expression of FASL mRNA correlates with AR, but is not detectable in non-rejected allografts. FASL has since been intensively explored as a potential marker for AR diagnosis, but the diagnostic accuracy is controversial and the applicability in clinical management is still unknown. Herein we document FASL using evidence-based medicine tools.

## Materials and Methods

### Literature search

Studies, of which FASL mRNA expression was evaluated as a diagnostic marker for post-transplant AR episodes, were identified by searching MEDLINE, EMBASE and Cochrane Library databases until February 2016 using following terms combination variously: “FASL or Fas ligand or FASLG or APTL or CD178 or CD95L or ALPS1B or CD95-L or TNFSF6 or TNLG1A or APT1LG1”, “rejection”, “renal transplant or kidney transplant or renal transplantation or kidney transplantation”. Reference lists of relevant articles were also checked manually to identify eligible studies.

### Eligibility criteria

Two reviewers (B.H. and Y.X.) independently identified relevant studies meeting the following inclusion criteria in duplicate: (1) assessed the diagnostic accuracy of FASL expression for AR episodes after kidney transplantation; (2) AR group and non-rejection group provided as two comparison groups; (3) FASL expression in peripheral blood (PBL), urine and/or allograft specimens were detected using qPCR methods; (4) Biopsy histopathological evaluation should be executed; and the diagnostic classification of AR and non-rejection on the basis of the Banff-97 criteria; (5) Data of SEN, SPE, and subjects numbers could be extracted to calculate the values of true negatives (TN), false negatives (FN), false positives (FP) and true positives (TP); (6) In each included studies, a housekeeping gene should be chosen as the reference gene compared to the expression levels of FASL, and the cut off values for AR diagnosis were set by the relative expression levels of FASL.

Studies only designated for pediatric recipients and other publication forms including reviews, letters, editorials, case reports and conference proceedings, were excluded.

### Data extraction

For all the studies, accuracy data form including TP, FP, FN, TN, first authors, countries, publication years, sample sources, quantitative methods, fluorescence staining, sample collecting time post renal transplantation, clinical classification of AR and the number of subjects, were extracted or calculated by two reviewers (C.L. and X.W.) independently. Non-rejection group included the subjects without any types of rejection based on Banff-97 criteria, while AR group contained any degrees of AR subjects.

### Quality assessment

The quality assessment of eligible studies in methodology were conducted by three reviewers (B.H., C.L. and L.S.) independently with the Quality Assessment of Diagnostic Accuracy Studies (QUADAS) tool, which includes 14 items and each item is assessed as ‘yes’, or ‘no’, or ‘unclear’. ‘yes’ scores 1 point, ‘no’ and ‘unclear’ score 0 point [13].

### Statistical analysis

This investigation was performed in accordance with the standard meta-analyses methods and procedures for diagnostic studies[14, 15]. Data were analyzed by Meta-DiSc 1.4, RevMan 5.0 and the Midas module in Stata 11.0 software[16]. We pooled the sensitivity (SEN), specificity (SPE), diagnostic odds ratio (DOR), positive likelihood ratios (PLR) and negative likelihood ratios (NLR) to estimate the usefulness of PCR techniques detecting FASL expression in AR diagnosis. The SEN/SPE of a test is defined as the proportion of people with/without disease who will have a positive/negative result, which are important measures of the diagnostic accuracy of a test[17]. The DOR is the ratio of the odds of positivity in disease relative to that in non-diseased, of which the value ranges from 0 to infinity and the high values indicate good test performance[18]. The likelihood ratios (LRs) indicate how much a given test would raise or lower the probability of having disease (PLR > 10 and NLR<0.1, 5<PLR<10 and 0.1<NLR<0.2, 2<PLR<5 and 0.2<NLR<0.5, indicate high, moderate, small diagnostic informativeness, respectively)[19, 20]. For a better clinical decision making direction, we calculated post-test probability of AR through combining the pre-test probability with the LRs using the Fagan’s nomogram. In this study, approximately 15% pre-test probability of AR in the first year post-renal transplant was chosen based on report of Brian et al. [1] and our clinical experience.

The summary receiver operating characteristic (SROC) curve was constructed to assess the diagnostic accuracy of a test and compare the usefulness of different tests[21], and the area under the SROC curve (AUC) provided a global diagnostic performance measure, values of which represented excellent (≥0.97), very good (0.93~0.96), and good (0.75~0.92) diagnostic accuracy, respectively[22].

I-square statistic was used to indicate the degree of heterogeneity problematic[15, 23]. statistically significant heterogeneity was considered if I-square > 50%[24]. To investigate the heterogeneity sources, we checked threshold effect by calculating the Spearman correlation coefficients, P<0.05 and an inverse correlation indicated a positive threshold effect [14]. The heterogeneity was also explored via the SEN analyses, meta-regression and subgroup analyses. SEN analyses were performed by sequential omission of individual studies to assess its influence on diagnostic performance. Publication bias was investigated by Deeks’ funnel plot, and P <0.05 for the slope coefficient indicated significant asymmetry[25].

## Results

### Study filtration

**Fig 1:**
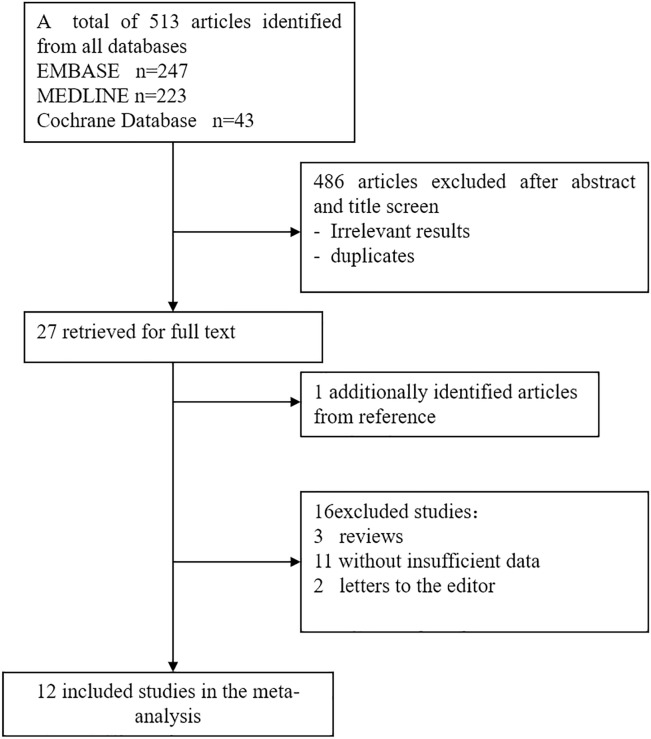
Study search flow for meta-analysis.

As the flow diagram (Fig 1) shows, 247, 223, and 43 relevant primary studies were identified by searching the MEDLINE, EMBASE, and Cochrane Library databases, respectively. After a carefully review and exclusion of studies considered to lack suitability, 12 studies published between 1997 and 2008 met the inclusion criteria[26–37]. Of the 12 studies, Lipman[27] and Dias et al.[32] evaluated the expression of molecular markers of AR in protocol biopsies of patients with and without subclinical AR; 2 studies[28, 31] detected the FASL expression in both graft biopsy and PBL samples, and 1 study [37] detected FASL in both urine and PBL samples. In the current study, we retrieved each group as an independent data set for a total of 15 data sets and 496 subjects. Table 1 outlines the characteristics of each study. For the assessment of methodologic quality, the average of QUADAS scores (range, 10 to 13) was approximately 11, demonstrating high-quality studies (S1 Table).

**Table 1:** Basic information extracted from the eligible studies.

Authors	Publication Year	Country	Sample	Method	Fluorescence Staining	Housekeeping Gene	Sample Collecting Time Post Tx	Number of Subjects	TP	FP	FN	TN
Strehlau et al.[26]	1997	US	graft biopsy	competitive RT-PCR	EB	GAPDH	day 4 and 11	40	10	2	2	26
Lipman et al.[27]	1998	US	graft biopsy	competitive RT-PCR	EB	GAPDH	1, 2, 3, and 6 months	21	2	0	9	10
Vasconcellos et al.[28]	1998	US	PBLs	competitive RT-PCR	EB	GAPDH	NA	31	11	5	0	15
Vasconcellos et al.[28]	1998	US	graft biopsy	competitive RT-PCR	EB	GAPDH	NA	31	9	4	2	16
Sharma et al.[29]	1998	US	graft biopsy	RT-PCR	EB	β-actin	NA	80	11	1	42	26
Dugre et al.[30]	2000	Canada	PBL	RT-PCR	EB	β-actin	3 months	21	2	1	6	12
Netto et al.[31]	2002	Brazil	PBL	RT-PCR	EB	β-actin	day 10	29	7	0	0	22
Netto et al.[31]	2002	Brazil	graft biopsy	RT-PCR	EB	β-actin	day 10	29	7	0	0	22
Dias et al.[32]	2004	Brazil	graft biopsy	RT-PCR	EB	GAPDH	2 months	21	8	1	3	9
Desvaux et al.[33]	2004	France	graft biopsy	Real-Time Quantitative RT-PCR	FHP	GAPDH	24biopsies:6–90 days; 10biopsies:5months-8years	41	23	0	9	9
Shin et al.[34]	2005	Korea	PBL	competitive RT-PCR	EB	β-actin	days 2, 4, 6, 8, 10, 12	15	1	0	6	8
Graziotto et al.[35]	2006	Italy	graft biopsy	Real-Time Quantitative RT-PCR	SYBR	GAPDH	diagnostic biopsy: 5–10days; protocol biopsy: 2 months	17	8	1	3	5
Galante et al.[36]	2006	Brazil	urine	Real-Time Quantitative RT-PCR	SYBR	cyclophilin	499 ± 629.6days	24	12	0	1	11
Dias et al.[37]	2008	Brazil	PBL	RT-PCR	FHP(TaqMan)	β-actin	7 to 55 days	48	18	2	2	26
Dias et al.[37]	2008	Brazil	urine	RT-PCR	FHP(TaqMan)	cyclophilin	7 to 55 days	48	20	10	0	18

FN, false negative; FP, false positive; PBL, peripheral blood; PCR, polymerase chain reaction; TN, true negative; TP, true positive; EB, ethidium bromide; FHP, fluorescence hybridization probes; Tx, renal transplant; NA, not available.

### Study Heterogeneity

S2 Table shows the results of SEN analysis. The greatest change in pooled DOR occurred when removing the data set from Sharma et al.[29], which changed from 30.63 to 35.90 (+17.20%). There was no change in the corresponding AUC value, suggesting that no single study could carry enough weight to significantly influence the pooled diagnostic performance of FASL for AR. In this meta-analysis, most of the I-squares in the overall pooled SEN (88.5%), SPE (63.6%), PLR (33.6%), NLR (92.5%), and DOR (13.7%) exceeded 50%, indicating substantial inter-study heterogeneity. The Spearman correlation coefficient was 0.215 (P = 0.441), suggesting that no threshold effect contributed to the heterogeneity. Furthermore, variables, including different area, publication year, sample source, quantitative method, fluorescence staining, sample collection time post-renal transplantation, and clinical classification of AR, may be the source of heterogeneity. Therefore, sub-group analyses were conducted.

### Overall diagnostic accuracy

**Fig 2:**
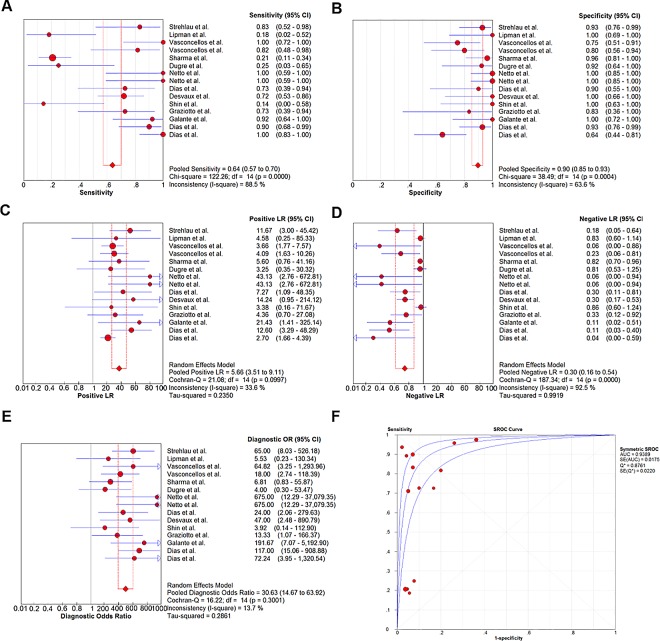
Diagnostic performance of FasL for AR. Forest plots of overall sensitivity(A), specificity(B), positive likelihood ratio (LR)(C), negative LR(D), diagnostic odds ratio (OR) (E), and SROC curve (F).

A random effects model was performed to investigate the overall diagnostic performance of FASL for AR. The overall pooled SEN, SPE, and DOR with a 95% confidence interval (CI) were 0.64 (0.57–0.70), 0.90 (0.85–0.93), and 30.63 (14.67–63.92), respectively. The AUC was 0.9389, indicating that FASL detection had very good diagnostic accuracy for AR (Fig 2).

**Fig 3:**
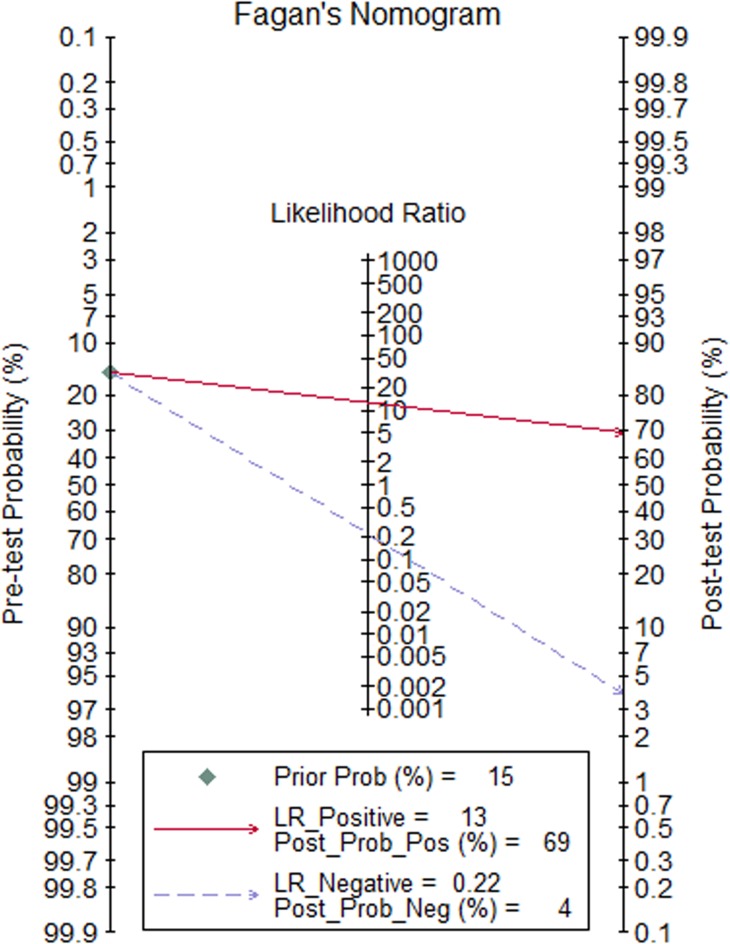
The Fagan's nomograms and post-test probability of FasL detection for developing AR.

Overall PLR syntheses (5.66; 95% CI, 3.51–9.11) showed that FASL had a moderate informational value in confirming AR, while the value of NLR (0.30; 95% CI, 0.16–0.54) had very small informational value for excluding AR (Fig 2). The Fagan's nomograms in this study demonstrated that the positive post-test probability was 69% and the negative post-test probability was 4%. Thus, for patients post-kidney transplantation, the probability of developing AR increased from 15% to 69% when FASL testing was positive, and reduced from 15% to 4% when FASL testing was negative (Fig 3).

### Sub-group analyses

**Fig 4:**
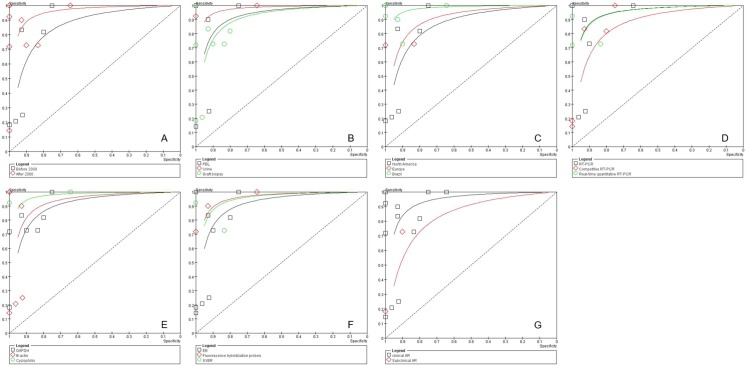
SROC plot of FasL test for AR diagnosis in subgroups analyses. (A), the year of publication; (B), sample; (C), area; (D), PCR technique; (E), housekeeping gene; (F), fluorescence staining; (G), clinical classification of AR.

Based on meta-regression, the p values of potential variables, such as the year of publication, sample origin, area, quantification method, housekeeping genes, clinical classification of AR, and fluorescence staining, were 0.2225, 0.6233, 0.7122, 0.7427, 0.4885, 0.7166, and 0.6905, respectively, all of which were > 0.05, indicating that none of the factors contributed to inter-study heterogeneity. The sub-group meta-analyses were also conducted and the results are presented in Fig 4 and Table 2.

**Table 2:** Diagnostic accuracy of FasL for AR after kidney transplantation.

Subgroups	No. of independent data sets	Independent estimates (95% CI)		Likelihood ratio (95% CI)		DOR (95% CI)	AUC (SE)
		SEN	SPE	PLR	NLR		
**Publication Year**							
Before 2000	6	0.43(0.33–0.52)	0.89(0.82–0.94)	4.51(2.76–7.35)	0.52(0.29–0.96)	16.06(6.18–41.74)	0.9149 (0.0354)
After 2000	9	0.81(0.73–0.88)	0.90(0.84–0.95)	8.70(3.34–22.64)	0.19(0.07–0.53)	56.44(21.50–148.13)	0.9526 (0.0185)
**Sample**							
PBL	5	0.74(0.60–0.85)	0.91(0.83–0.96)	6.27(2.48–15.86)	0.27(0.07–1.08)	34.71(5.64–213.66)	0.9561 (0.0237)
Urine	2	0.97(0.84–1.00)	0.74(0.58–0.87)	5.71(2.02–5.44)	0.09(0.02–0.33)	110.64(12.50–979.34)	SROC-Unavailable
Graft biopsy	8	0.53(0.44–0.61)	0.93(0.88–0.97)	6.41(3.58–11.49)	0.37(0.19–0.71)	22.42(9.44–53.23)	0.9264 (0.0315)
**Area**							
North America	6	0.43(0.33–0.52)	0.89(0.82–0.94)	4.51(2.76–7.35)	0.52(0.29–0.96)	16.06(6.18–41.74)	0.9149 (0.0354)
Europe	2	0.72(0.56–0.85)	0.93(0.68–1.00)	6.31(1.39–28.69)	0.31(0.19–0.50)	22.75(3.35–154.47)	SROC-Unavailable
Brazil	6	0.92(0.84–0.97)	0.89(0.82–0.94)	11.21(3.00–41.94)	0.14(0.07–0.28)	108.44(33.88–347.13)	0.9648 (0.0158)
**Quantitative Technique**							
RT-PCR	7	0.58(0.49–0.67)	0.90(0.84–0.94)	7.57(2.77–20.72)	0.22(0.06–0.78)	40.56(10.19–161.41)	0.9534 (0.0207)
Competitive RT-PCR	5	0.63(0.49–0.76)	0.87(0.78–0.93)	4.48(2.69–7.47)	0.38(0.14–1.06)	22.57(7.43–68.52)	0.9168 (0.0351)
Real-time quantitative RT-PCR	3	0.77(0.64–0.87)	0.96(0.80–1.00)	8.43(2.25–31.64)	0.28(0.17–0.45)	38.93(7.43–204.06)	0.8874 (0.1543)
**Housekeeping gene**							
GAPDH	7	0.72(0.62–0.80)	0.87(0.79–0.93)	4.81(2.99–7.73)	0.30(0.13–0.68)	26.07(10.30–65.96)	0.9158 (0.0304)
β-actin	6	0.45(0.35–0.55)	0.97(0.92–0.99)	10.21(4.35–23.92)	0.42(0.18–0.99)	31.79(5.29–191.18)	0.9845 (0.0121)
Cyclophilin	2	0.97(0.84–1)	0.74(0.58–0.87)	5.71(0.50–65.85)	0.09(0.02–0.33)	110.64(12.50–979.34)	SROC-Unavailable
**Fluorescence Staining**							
EB	9	0.49(0.41–0.58)	0.92(0.87–0.96)	5.21(3.31–8.21)	0.43(0.24–0.78)	22.05(8.39–57.94)	0.9358(0.0251)
FHP	3	0.85(0.74–0.92)	0.82(0.70–0.90)	6.43(1.33–31.12)	0.14(0.03–0.68)	82.92(19.34–355.51)	0.9575(0.0233)
SYBR	3	0.83(0.63–0.95)	0.94(0.71–1)	7.22(1.54–33.83)	0.22(0.07–0.67)	40.58(3.07–535.85)	SROC-Unavailable
**clinical classification of AR**							
clinical AR	13	0.66(0.59–0.72)	0.89(0.85–0.93)	5.84(3.43–9.94)	0.24(0.11–0.52)	34.85(15.34–79.17)	0.9400(0.0177)
Subclinical AR	2	0.46(0.24–0.68)	0.95(0.75–1)	6.35(1.29–31.11)	0.54(0.15–1.93)	13.81(1.99–96.00)	SROC-Unavailable
**Sample Collecting Time Post Tx (<6months)**	10	0.73(0.64–0.81)	0.90(0.85–0.94)	6.90(3.24–14.69)	0.30(0.13–0.67)	33.62(12.08–93.61)	0.9486(0.0193)
**Overall**	15	0.64(0.57–0.70)	0.90(0.85–0.93)	5.66(3.51–9.11)	0.30(0.16–0.54)	30.63(14.67–63.92)	0.9389(0.0175)

AUC, the area under the summary receiver operating characteristics curve; CI, confidence interval; DOR, diagnostic odds ratio; NLR, negative likelihood ratio; PLR, positive likelihood ratio; PBL, peripheral blood; SEN, sensitivity; SPE, specificity; Tx, renal transplantation, EB, ethidium bromide; FHP, fluorescence hybridization probes.

#### Publication year

According to the publication year, all data sets were divided into sub-groups of the year before (including studies published in 2000) and after 2000. The pooled PLR, NLR, and DOR of the year before the 2000 sub-group were 4.51, 0.52, and 16.06, respectively, while in the year after the 2000 sub-group were 8.70, 0.19, and 56.44, respectively (Fig 4A).

#### Sample source

Samples in the included studies were obtained from allograft biopsy tissues, PBL, or urine. The pooled DOR values for the graft biopsy, PBL, and urine subgroups were 22.42, 34.71, and 110.64, respectively. The pooled PLRs were 6.27, 5.71, and 6.41, respectively. The pooled NLRs were 0.27, 0.09, and 0.37, respectively (Fig 4B).

#### Area

We also divided the data into four sub-groups according to the country, as follows: North America (7 studies); Europe (2 studies); Brazil (6 studies); and Korea (1 study). The pooled DOR values for the North America, Brazil, and Europe sub-groups were 16.06, 108.44, and 22.75, respectively. The pooled PLRs were 4.51, 6.31, and 11.21, respectively. The pooled NLRs were 0.52, 0.31, and 0.14 (0.07–0.28), respectively (Fig 4C).

#### Quantitative technique

Three PCR techniques were used to quantify FASL, as follows: 8 studies used reverse transcriptase PCR (RT-PCR); 6 studies used competitive RT-PCR; and 5 studies used real-time quantitative RT-PCR. The pooled PLRs, NLRs, and DORs of the FASL analysis were 7.57, 0.22, and 40.56 for the RT-PCR sub-group, respectively, 4.48, 0.38, and 22.57 for the competitive RT-PCR sub-group, respectively, and 8.43, 0.28, and 38.93 for the real-time quantitative RT-PCR sub-group, respectively (Fig 4D).

#### Housekeeping genes

Three housekeeping genes were used as reference genes to measure the relative level of FASL mRNA expression, as follows: GAPDH (7 studies); β-actin (6 studies); and cyclophilin (2 studies). The pooled PLRs, NLRs, and DORs were 4.81, 0.30, and 26.07 for the GAPDH sub-group, respectively, 10.21, 0.42, and 31.79 for the β-actin sub-group, respectively, and 5.71, 0.09, and 110.64 for the cyclophilin sub-group, respectively (Fig 4E).

#### Fluorescence staining

There were three different fluorescence staining techniques used amongst the included studies to quantify FASL mRNA expression, as follows: ethidium bromide (EB; 9 studies); fluorescence hybridization probes (3 studies); and SYBR (3 studies). The pooled PLRs, NLRs, and DORs were 5.21, 0.43, and 22.05 for the EB sub-group, respectively, 6.43, 0.14, and 82.92 for the fluorescence hybridization probe sub-group, respectively, and 7.22, 0.22, and 40.58 for the SYBR sub-group, respectively (Fig 4F).

#### Sample collection time post-renal transplantation (< 6 months)

To evaluate the value of FASL detection for diagnosing AR during the early post-transplant period, 8 studies (10 data sets) in which samples were collected within 6 months were extracted and independently analyzed. The pooled PLRs, NLRs, and DORs were 6.90 (3.24–14.69), 0.30 (0.13–0.67), and 33.62 (12.08–93.61), respectively.

#### Clinical classification of AR

The clinical and sub-clinical AR groups were analyzed. The pooled PLRs, NLRs, and DORs were 6.35, 0.54, and 13.81 for the sub-clinical AR sub-group, respectively, and 5.84, 0.24, and 34.85 for the clinical AR sub-group. Moreover, the SPE (0.95) was high in the sub-clinical AR sub-group (Fig 4G).

The SROC plot of sub-group analyses showed that detection of FASL in the urine by real-time quantitative RT-PCR (cyclophilin as a housekeeping gene) may be a better choice for AR diagnosis.

### Comparing the diagnostic performance of granzyme B, perforin, and FASL

**Fig 5:**
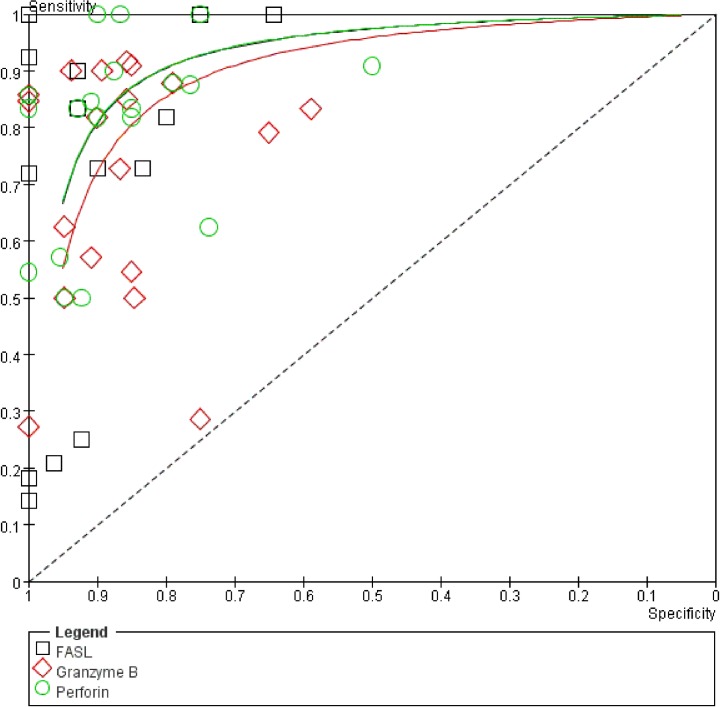
SROC plot of FasL, granzyme B and perforin for AR diagnosis.

To identify a better diagnostic biomarker for AR, SEN and SPE from the included studies and our previous meta-analysis data of PCR testing, perforin and granzyme B [38] were plotted in a visualized SROC plot to compare FASL, perforin, and granzyme B (Fig 5). Forest plots of the three makers for AR showed wide variation in SEN, ranging from 25%-100%, 50%-100%, and 27%-92%, respectively (S1 Fig), and the SPE varied from 64%-100%, 50%-100%, and 59%-100%, respectively (S1 Fig). FASL testing had better diagnostic performance when compared to perforin (AUC = 0.9123) and granzyme B (AUC = 0.8858; Fig 5).

### Publication bias

**Fig 6:**
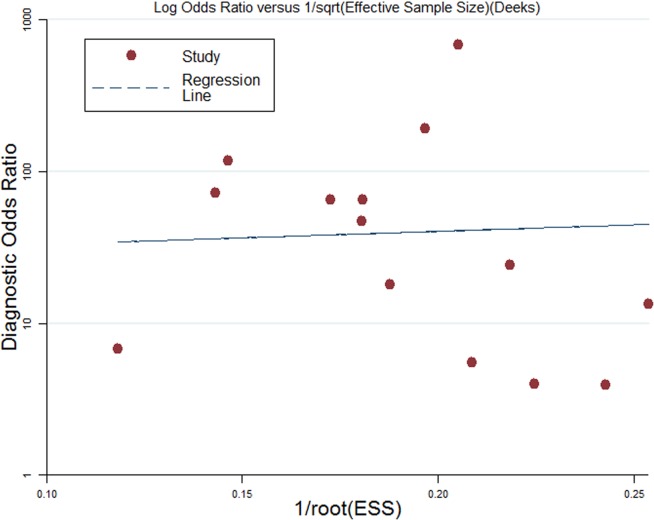
Deeks’ funnel plot for the assessment of potential bias in FasL analysis.

In the overall FasL analyses, a Deeks’ funnel plot was created; no asymmetric distribution was apparent (Fig 6). The P value for the slope coefficient was 0.862 (P >0.05), indicating no evident publication bias.

## Discussion

Data were pooled from 496 subjects in 12 studies in the current meta-analysis with the goal to demonstrate whether or not FasL is a reliable immune marker for diagnosing AR when applied to clinical management for renal transplant recipients. Based on the QUADAS scores, all of the 12 included studies had good quality. In the overall analysis, SEN was relatively poor in diagnosing AR [0.64 (0.57–0.70)], while SPE had good diagnostic value [0.90 (0.85–0.93)]. According to the guideline, the overall AUC (0.9389) had very good global diagnostic performance[22]. A test for heterogeneity showed that inter-study heterogeneity existed in the current study, without a threshold effect. In the included studies, various factors (different area, publication year, sample source, quantitative method, fluorescence staining, and clinical classification of AR) may have been the source of heterogeneity; however, the meta-regression analysis showed that none of the variables significantly led to inter-study heterogeneity. The AUC in PBL sub-group analysis (0.9561), which indicated very good diagnostic performance, was higher than the graft biopsy sub-group (0.9264); FASL mRNA was detected in the urine, in which cyclophilin was used as the housekeeping gene and had the best DOR value (110.64).

Because the SEN and SPE of a test cannot be used to estimate the probability of disease in individual patients, LRs are more clinically meaningful [39]. We explored LR syntheses in this meta-analysis; however, only sub-groups after 2000 (PLR, 8.70 > 5; NLR, 0.19 < 0.2), Brazil (PLR, 11.21 > 5; NLR, 0.14 < 0.2), fluorescence hybridization probes (PLR, 6.43 > 5; NLR, 0.14 < 0.2), and urine ([also the cyclophilin] PLR, 5.71 > 5; NLR, 0.09 < 0.2) suggested moderate informational diagnostic value. The overall and other sub-group LR syntheses had small diagnostic informative value. Furthermore, the individual post-test probability, which is the likelihood of having the disease (AR), was estimated by using LRs in conjunction with the pre-test probability of the disease (15%) [1] and presented on Fagan’s nomogram for further routine clinical practice [40]. In the current study, the post-test probability of positive FASL detection (69%) may result in clinicians taking percutaneous needle biopsy surveillance and more therapeutic intervention into consideration. Conversely, if FASL detection is negative (post-test probability, 4%), the likelihood of developing AR in recipients is so small that percutaneous needle biopsy is not recommended for surveillance and further diagnosis.

Apoptosis effected by CTLs, thought to play a major role in renal allograft rejection, is mediated by two major effector pathways (the Fas-FASL lytic pathway and the perforin/granzyme B degranulation pathway) [41]. In earlier studies, there was a focus on the manipulation of tissues to express FASL, which may be feasible for preventing graft rejection. Bellgrau et al.[42] suggested that FASL can be used to create immune-privileged tissues for a variety of transplant uses, indicating that FASL may play an important role in preventing graft rejection. Subsequent research[43] showed that the FASL signal could provide site- and immune-specific protection of islet allografts. Seino et al.[44] first reported that FASL, as a factor independent of Fas in the grafts, contributes substantially to cardiac allograft rejection. Seino et al. [45]then suggested that the kidney capsule is an appropriate site for establishing FASL-mediated immune privilege, but prior to application in humans, further verification of the Fas/FASL system *in vivo* is necessary. In 1996, Sharma et al.[12] identified the differential expression of the two major lytic pathways (FASL and Fas; and granzyme B and perforin) in acute and chronic allograft rejection and suggested that specific therapy directed at the cytotoxic attack molecules might be efficacious in the prevention and/or treatment of AR. Sharma et al.[12] showed that FASL mRNA expression of human renal allografts correlated with AR, but not chronic rejection, and FASL mRNA expression may not exist in non-rejected allografts. For the first time, the study explored the intrarenal expression of FASL mRNA for AR diagnosis and showed a high SPE (96%), but the SEN was only 21%. A subsequent investigation of renal allografts supported the notion that comprehensive molecular analyses of allograft tissues can provide predictive, diagnostic, and prognostic information with respect to human allograft rejection[29]. Thereafter, clinicians pay more attention on this promising immune strategy around rejection. Strehlau et al.[26] suggested that combined intrarenal gene expression analysis of CTL effector molecules (FASL, perforin, and granzyme B) by quantitative RT-PCR provides a rapid and reliable tool for AR diagnosis and follow-up, with extraordinary SEN (100%) and SPE (100%). Desvaux et al.[33] also suggested that the simultaneous measurement of the FASL and granzyme B mRNA over-expression by real-time RT-PCR quantification in kidney graft biopsies might represent an efficient new tool for the prediction of pejorative outcomes of AR. Lipman et al. [27] reported that the amount of CTL effector molecule transcripts, including FASL, perforin, and granzyme B, exist in biopsies with sub-clinical AR, thus demonstrating that rejection is often accompanied by enhanced expression of pro-inflammatory gene transcripts despite the absence of overt graft dysfunction. As this state of sub-clinical rejection could prove detrimental to long-term graft function, a role for surveillance biopsies of stable grafts with intent-to-treat sub-clinical rejection should be considered. This conclusion was supported by Dias et al.[32], who showed that FASL expression has a higher SPE (88%) than granzyme B and perforin for the diagnosis of sub-clinical AR 12 months after kidney transplantation. Herein we synthesized data investigating sub-clinical AR, and showed a high pooled SPE (0.95), but a poor SEN (0.46). The pooled DOR (13.81) was clearly lower than the clinical AR sub-group (34.85), which limited the utility in AR surveillance.

Furthermore, it is difficult to recommend surveillance biopsies in clinical practice due to the invasiveness. The role of non-invasive monitoring via plasma or urine biomarkers has been a topic of interest to the transplant community [46]. Vasconcellos et al. [28] reported that FASL gene expression in PBL is closely associated with the pathologic diagnosis of rejection, which may serve as a non-invasive method for monitoring AR. Moreover, simultaneous up-regulation of any two of the three CTL effector molecules (granzyme B, perforin, and FASL) gene expression in PBL could diagnose AR with extraordinary SEN and SPE. Dugre et al.[30] shared a similar view. The evaluation of cytotoxic molecules has proved useful in the clinical identification of post-transplant AR and in the justification of concomitant anti-rejection therapy before histologic diagnosis confirmation. Furthermore, Netto et al.[31] compared granzyme B, FASL, and perforin gene expression during AR episodes after renal transplant between PBL and renal aspirates, and found that expression of the three CTL effector molecules in PBL is sufficient for diagnosing AR. In contrast, Shin et al.[34] did not identify a consistent expression pattern of granzyme B and FASL associated with rejection. Graziotto et al.[35] also showed that FASL expression in PBL does not correlate with histologic AR. FASL, as a non-invasive surrogate marker for biopsies in AR surveillance and diagnosis, is controversial. Galante et al.[36] analyzed FASL in urine by real time RT-PCR and flow cytometry, and showed high SEN (91% and 88%, respectively) and SPE (100% for each) for AR, suggesting that both techniques are equally useful for non-invasive monitoring kidney allografts. Dias et al.[37] showed that quantification of the FASL gene in PBL and urinary cells from kidney transplant recipients with DGF may provide a useful and accurate non-invasive diagnosis of AR. In the current meta-analysis, we showed that FASL detection using the non-invasive method (PBL and urine), especially urine, have better test performance than kidney graft biopsies.

In the multicenter Clinical Trials in Organ Transplantation 04 (CTOT-04) study, Suthanthiran et al. [46] showed that the AUC for the combination of 18S-normalized perforin mRNA, 18S-normalized IP-10 mRNA, and 18S rRNA in urinary cells to diagnosis ACR was 0.84, which was only slightly lower than the final concluded diagnostic signature of CD3ε mRNA, IP-10 mRNA, and 18S rRNA (AUC, 0.85). The inclusion of granzyme B and/or perforin did not significantly improve the diagnostic signature based on IP-10, CD3ε, and 18S, due largely to the high correlation between the levels of CD3ε mRNA and granzyme B or perforin mRNA. Suthanthiran et al. [46] also suggested that 18S-normalized levels of granzyme B and perforin are strongly associated with ACR when considered alone. The CTL effector molecules serve as promising urinary biomarkers in predicting the risk of ACR in kidney transplant recipients. Herein we also compared the diagnostic performance of FASL with granzyme B and perforin; the diagnostic accuracy for AR after kidney transplantation was summarized in our previous study[47]. The results showed that FASL has a better test performance than granzyme B and perforin.

Quantitative PCR, which was first introduced in 1992 by Higuchi et al. [48], has been a common technique in basic and clinical transplantation research [49] and is considered to be the most accurate and reliable technique in validating data obtained by other methods [50]. Reference genes are an internal reaction control that have sequences which differ from the target. Of the three reference genes in the current meta-analysis, GAPDH and β-actin were most commonly used; in two studies involving the urine sub-group, cyclophilin was used as a reference gene, which showed a high pooled DOR. Cyclophilin is the primary cytosolic receptor for cyclosporin A (CsA). The high affinity of cyclophilin for CsA and specificity for immunosuppressive cyclosporine analogs implicates cyclophilin as a pivotal regulator of T and B cell activation [51]. Cyclophilin binding has been reported to be a more accurate measure of cyclosporine immunosuppressive activity after renal transplantation [52]. Among known human cyclophilins, cyclophilin A as a housekeeping protein is the most abundant cytosolic member, which can be secreted into the extracellular environment in various cell types due to inflammatory stimuli[53]. Cyclophilin, as a reference gene, normalizes FASL mRNA expression and may reflect AR better than GAPDH and β-actin. Moreover, the technique of fluorescence hybridization probes in qPCR showed a better diagnostic accuracy than EB and SYBR.

The current meta-analysis is the first study to summarize the diagnostic performance of FASL mRNA expression for AR after kidney transplantation. Like other meta-analyses, the main weaknesses of the present study stem from the limitations of the included studies. First, the overall inference may be clouded when pooling the urine, graft, and PBL data because clear differences and minimal overlap in gene expression patterns between kidney biopsy tissues and circulating lymphocytes of AR patients have been reported[54]. FASL mRNA expression is up-regulated in the urine, grafts, and PBL during AR episodes, as reported in the included studies, but the differential expression in the three sample sources are still unclear, though clearly high DORs occur in the urine. Analysis of the amounts of gene transcripts in the qPCR technique is based on relative calculations and reference gene normalization differed in the included studies, and no research has been specifically designed for that comparison. In the current analysis, significant heterogeneity existed and the source is still unknown, even though we conducted SEN, sub-group, and meta-regression analyses. Differences in the patient population, sample size, and research design may have been contributing factors in the included studies. Second, we failed to identify a universal cut-off value for FASL mRNA expression for AR because of the different reference normalization. Third, in the current meta-analysis, only 7 studies explored FASL as a non-invasive diagnostic marker and 2 studies focused on sub-clinical AR episodes. In addition, the included studies of this meta-analysis were all published before 2008(including the year of 2008), and there are no recent biomarker studies focused on FASL, new studies need to be conducted to clarify the clinical applicability of FASL.

In conclusion, data from the current meta-analysis suggest the diagnostic potential for FASL mRNA detection as a reliable immune marker of AR in renal allograft recipients. Additional large, multicenter, prospective studies are needed to validate the power of this test marker in the non-invasive diagnosis of AR after renal transplantation, especially when combined with other candidate biomarkers.

## Supporting Information

S1 Fig Forest plots of sensitivity and specificity of PCR test detecting granzyme B, perforin, and FasL for AR diagnosis. Tests with “b”, “u”, “p” mean the sample originate from graft biopsy, urine, and peripheral blood, respectively.
(TIF)

S1 PRISMA Checklist PRISMA Checklist. (DOC)

S1 Table Quality assessment. (DOC)

S2 Table The influence of each study for the test accuracy of FasL. (DOC)
