# Supplementary figures and images for: Diagnostic Performance of Fas Ligand mRNA Expression for Acute Rejection after Kidney Transplantation: A Systematic Review and Meta-Analysis
Source: PLoS One. 2016 Nov 3;11(11):e0165628. doi: 10.1371/journal.pone.0165628 (PMC5094747; doi:10.1371/journal.pone.0165628)

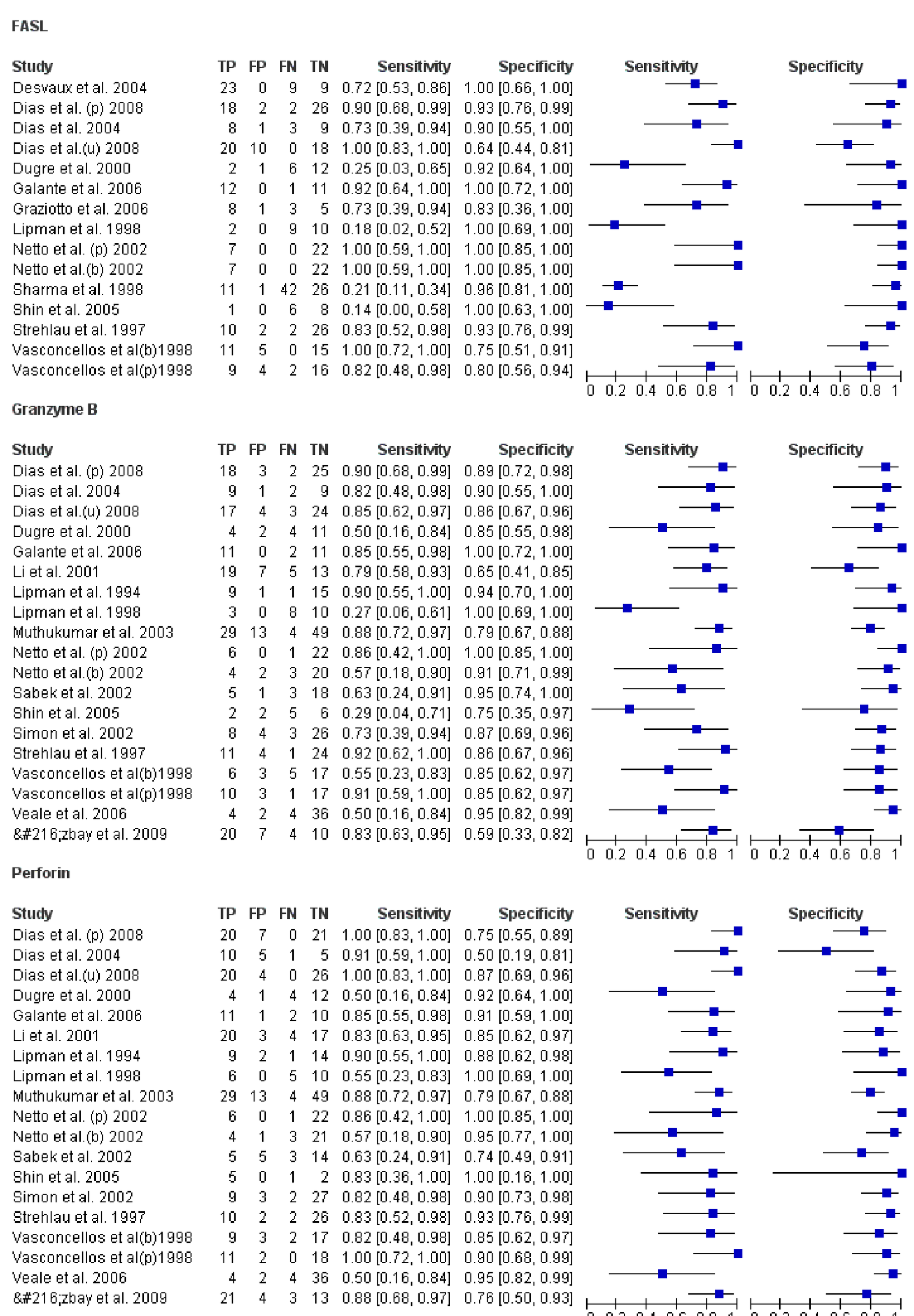

Supplement: S1 Fig — Tests with “b”, “u”, “p” mean the sample originate from graft biopsy, urine, and peripheral blood, respectively. (TIF) [file pone.0165628.s001.tif]
