# Supplementary material for: Diagnostic Performance of Fas Ligand mRNA Expression for Acute Rejection after Kidney Transplantation: A Systematic Review and Meta-Analysis
Source: PLoS One. 2016 Nov 3;11(11):e0165628. doi: 10.1371/journal.pone.0165628 (PMC5094747; doi:10.1371/journal.pone.0165628)
Supplement: S2 Table — (DOC) [file pone.0165628.s004.doc]

**S2 Table. The influence of each study for the test accuracy** of FasL

| **Author** | **StudyId** | **DOR** | **change（DOR）** | **AUC** | **change(AUC)** |
| --- | --- | --- | --- | --- | --- |
| Strehlau et al. | 1997 | 28.394 | -7.30% | 0.9353 | -0.38% |
| Lipman et al. | 1998 | 33.425 | 9.13% | 0.939 | 0.01% |
| Vasconcellos et al. | 1998 | 29.518 | -3.63% | 0.9419 | 0.32% |
| Vasconcellos et al. | 1998 | 33.036 | 7.86% | 0.9484 | 1.01% |
| Sharma et al. | 1998 | 35.899 | 17.20% | 0.9389 | 0.00% |
| Dugre et al. | 2000 | 35.166 | 14.81% | 0.9401 | 0.13% |
| Netto et al. | 2002 | 27.576 | -9.97% | 0.9347 | -0.45% |
| Netto et al. | 2002 | 27.576 | -9.97% | 0.9347 | -0.45% |
| Dias et al. | 2004 | 31.533 | 2.95% | 0.9398 | 0.10% |
| Desvaux et al. | 2004 | 30.087 | -1.77% | 0.9375 | -0.15% |
| Shin et al. | 2005 | 33.507 | 9.39% | 0.9391 | 0.02% |
| Graziotto et al. | 2006 | 32.886 | 7.37% | 0.9431 | 0.45% |
| Galante et al. | 2006 | 28.059 | -8.39% | 0.9353 | -0.38% |
| Dias et al. | 2008 | 26.067 | -14.90% | 0.9318 | -0.76% |
| Dias et al. | 2008 | 29.238 | -4.54% | 0.9453 | 0.68% |
| Overall |  | 30.63 |  | 0.9389 |  |

AUC, the area under the summary receiver operating characteristics curve; DOR, diagnostic odds rate.
